# Reconstructive Liposuction for Residual Lipodystrophy After Remission of Cushing’s Disease: A Case Report

**DOI:** 10.7759/cureus.86794

**Published:** 2025-06-26

**Authors:** Emilio Mondragón Rosas, José E González Flores, Ricardo J Mondragón Zepeda, Andrea L González Muñóz, Ana D Zamudio Carías, Pablo E Navarro López, Airam A Arias Villaverde, Colin A Ramirez Díaz, Michelle Cruz Méndez, Lourdes Rivas Ayala

**Affiliations:** 1 School of Medicine and Health Sciences, Tecnológico de Monterrey (ITESM), Mexico City, MEX; 2 Plastic and Reconstructive Surgery, Centro Médico Nacional La Raza, Mexico City, MEX; 3 Psychology, Tecnológico de Monterrey (ITESM), Mexico City, MEX; 4 Medicine, Benemérita Universidad Autónoma de Puebla (BUAP), Puebla City, MEX

**Keywords:** acth-secreting pituitary adenoma, cushing’s syndrome, lipodystrophy, liposuction, psychological burden, social functioning

## Abstract

Cushing’s syndrome (CS) is often presented due to an adrenocorticotropic hormone (ACTH)-secreting pituitary adenoma, characterized by high chronic cortisol levels. Surgical resection of the pituitary adenoma is the primary treatment, but long-term metabolic and physical sequelae can persist, affecting psychological well-being and social functioning. Glucocorticoids are directly involved in alterations of fat metabolism, favoring centripetal adiposity. Even after hormonal normalization, patients may experience residual lipodystrophy. Impairment of body image may cause psychological distress and social isolation. The objective is to illustrate the potential therapeutic value of reconstructive liposuction in restoring body image and psychological well-being in a patient with persistent lipodystrophy after Cushing’s disease remission.

We report a case of a 16-year-old female with recurrent Cushing’s disease secondary to a pituitary microadenoma, confirmed by elevated urinary free cortisol and magnetic resonance imaging (MRI). It was initially treated with transsphenoidal resection in 2019; disease recurrence was confirmed and again treated in 2024. Despite intervention, the prolonged hypercortisolism developed into secondary lipodystrophy, leading to severe body image dissatisfaction and social withdrawal. Thyroid function remained euthyroid, ruling out metabolic contributors. Because of the psychological distress caused by persistent fat redistribution, the patient underwent elective liposuction in 2025. Postoperative follow-up revealed reduced psychological distress and improved well-being and self-esteem. Reconstructive liposuction can play a key role in the treatment and management of persistent post-CS lipodystrophy, contributing significantly to psychological recovery. Prospective studies evaluating surgical criteria and long-term psychosocial outcomes are needed to define eligibility criteria and assess outcomes, leading to the development of clinical guidelines for aesthetic interventions in post-CS recovery.

## Introduction

Corticotroph pituitary adenomas (corticotropinomas) are pituitary tumors that secrete excess adrenocorticotropic hormone (ACTH), causing endogenous Cushing's syndrome (CS). Most of these adenomas are sporadic and monoclonal, although in some rare cases, they are associated with germline mutations (e.g., in USP8) or genetic syndromes [1,2]. Clinically, excess ACTH causes a classic presentation with centripetal obesity, purple striae, muscle asthenia, hypertension, and emotional disturbances such as depression or anxiety [3-5]. Chronically elevated cortisol levels promote fat deposition in central body regions - face, neck, torso, and abdomen - at the expense of relative thinning of the limbs [3], leading to lipodystrophy that can seriously affect the patient's quality of life.

At the molecular level, glucocorticoids stimulate the differentiation of preadipocytes into mature adipocytes and enhance lipoprotein lipase activity in peripheral fat tissues [6], thereby increasing the uptake of circulating fatty acids and the storage of triglycerides. At the same time, they increase hepatic lipogenesis and modulate cortisol receptor homeostasis (e.g., 11β-HSD1 in adipose tissue), favoring visceral fat distribution [6]. Although glucocorticoids can induce acute lipolysis, they exert chronic lipogenic effects - especially in subcutaneous adipose tissue - which promotes fat accumulation in the face, neck, and trunk [6]. This central adiposity, characteristic of CS, is further enhanced by increased hepatic lipogenesis and the overexpression of 11β-HSD1 in adipose tissue, which amplifies the local action of cortisol [6].

## Case presentation

**Figure 1:**
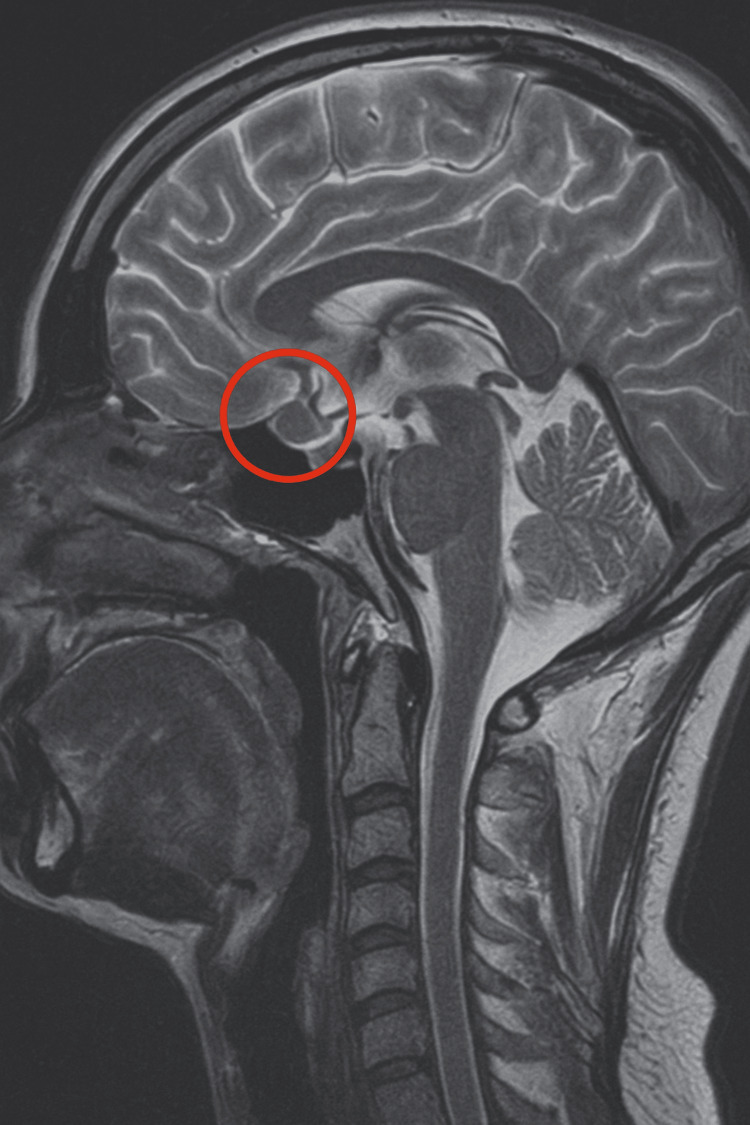
T1-weighted sagittal MRI scan showing a corticotroph pituitary microadenoma (4 × 3 mm) circled in red The lesion is localized within the anterior pituitary gland, consistent with an ACTH-secreting adenoma causing Cushing’s disease in the patient. MRI, magnetic resonance imaging; ACTH, adrenocorticotropic hormone

In 2019, a 16-year-old female patient was initially diagnosed with a 4 × 3 mm pituitary microadenoma (Figure 1), following clinical suspicion of Cushing’s disease. The diagnosis was confirmed through imaging studies and endocrinological testing, which revealed consistently elevated urinary free cortisol levels ranging from 459 to 740.07 µg/24 hours (normal range: <50 µg/24 hours), indicative of endogenous hypercortisolism. No dynamic load tests (such as dexamethasone suppression or ACTH stimulation) were performed, as the diagnosis was supported by the clinical context and laboratory findings. Moreover, no clinical or biochemical evidence of adrenal insufficiency was observed during follow-up.

**Figure 2:**
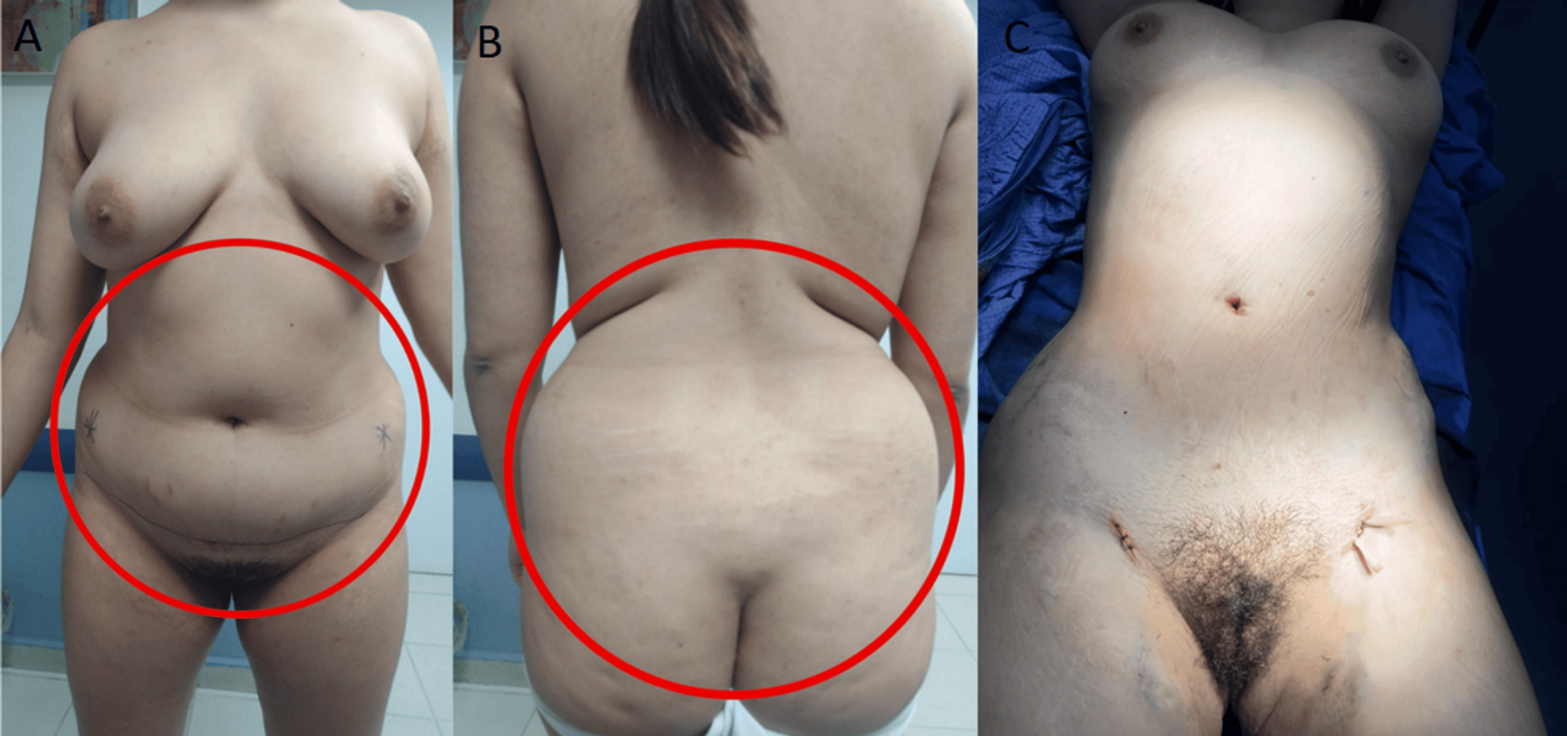
Preoperative and intraoperative images of the patient A and B panels show the anterior and posterior views prior to liposuction, demonstrating centripetal adipose accumulation characteristic of Cushing’s syndrome. The C panel shows the intraoperative stage following abdominal and flank liposuction, with placement of drainage tubes, and visible reduction in subcutaneous fat volume.

The patient underwent transsphenoidal endonasal resection of the pituitary tumor in 2019. Although initially successful, disease recurrence was confirmed, and a second endonasal transsphenoidal surgery was performed in 2024. Despite these interventions, the prolonged hypercortisolism led to the development of secondary lipodystrophy, manifesting as centripetal fat accumulation, a dorsal fat pad, and disproportionate truncal adiposity (Figure 2). These physical alterations had a significant psychosocial impact, as reported by the patient during follow-up visits, resulting in body image dissatisfaction, low self-esteem, and social withdrawal. No formal psychometric scales were administered.

A thyroid function panel revealed a slightly elevated thyroid-stimulating hormone (TSH) level (4.280 μUI/mL; reference range: 0.270-4.200), with total and free T3 and T4 values within normal limits, ruling out clinically significant hypothyroidism as a confounding factor for her phenotype. The biochemical profile suggested a euthyroid state, despite borderline TSH elevation, which was interpreted as a subclinical or adaptive response to chronic cortisol excess (Table 1).

**Table 1:** Comparison between the patient’s hormone levels and standard reference ranges A persistently elevated 24-hour urinary cortisol range is observed, consistent with endogenous hypercortisolism. The thyroid profile remains within normal limits, with a mildly elevated TSH in the absence of overt thyroid dysfunction. These findings support the functional and metabolic profile characteristic of Cushing’s syndrome. TSH, thyroid-stimulating hormone

Parameter	Normal Range	Patient’s Value
Cortisol (µg/24 hour)	58.0 - 403.0	459.5 - 740.07
TSH (µUI/mL)	0.270 - 4.200	4.280
Total T3 (ng/mL)	0.80 - 2.00	1.02
Free T3 (pg/mL)	2.00 - 4.40	3.33
Total T4 (µg/dL)	4.50 - 12.00	8.63
Free T4 (ng/dL)	0.92 - 1.68	1.36

The procedure targeted lipodystrophic regions identified through clinical examination and patient concerns, rather than formal imaging or anthropometric measurements. It aimed to restore body contour, alleviate somatic distress, and improve her overall self-perception and quality of life. Postoperative follow-up revealed patient-reported improvements in body image and psychological well-being. While these outcomes were not evaluated with formal instruments, the clinical improvement was evident and significant from the patient’s perspective, highlighting the role of plastic surgery not only as a reconstructive tool, but also as a therapeutic strategy for restoring dignity and social functioning in patients recovering from CS.

## Discussion

After successful treatment of the pituitary adenoma, many metabolic parameters improve; however, fat distribution usually only partially reverses. Longitudinal studies show that, in the medium term, weight and abdominal circumference decrease, and there is some redistribution of fat toward the limbs following cortisol remission [3].

For example, Bavaresco et al. (2024) observed that, after hormone levels normalized, total fat was reduced and part of it shifted from the visceral area to the legs [3]. Nevertheless, their review highlights that a significant proportion of patients continue to present with residual visceral adiposity and moderate obesity (body mass index, or BMI >25), despite hormonal control [7]. In our case, truncal adiposity persisted based on clinical assessment, though no formal anthropometric measurements were performed.

Although liposuction is not traditionally considered first-line therapy for cortisol-induced lipodystrophy secondary to Cushing's disease, increasing evidence from related lipodystrophic syndromes supports its clinical utility. For instance, in human immunodeficiency virus (HIV)-associated cervicodorsal lipodystrophy, Barton et al. (2021) conducted a 15-year retrospective analysis comparing liposuction and excisional lipectomy, finding that 80% of patients undergoing liposuction alone experienced recurrence, while none of the patients treated with excisional lipectomy showed recurrence - albeit with a higher risk of postoperative seroma formation [7]. These findings underscore that, while liposuction may be less durable than excision, it remains a viable option for selected cases, especially when used for contouring or as an adjunct [7]. Similarly, the Endocrine Society guidelines on lipodystrophy management emphasize the importance of personalized approaches, particularly when localized adipose accumulation contributes to persistent metabolic dysfunction or psychological distress [8]. Akinci et al. (2024) also highlight that, even in partial or atypical lipodystrophy syndromes, patients often report substantial impairment in quality of life due to disfiguring fat redistribution [9]. In this context, liposuction should not be dismissed as merely cosmetic but considered part of a functional and psychosocial rehabilitation strategy. The present case exemplifies this rationale, as the patient - despite biochemical remission of Cushing’s disease - continued to experience debilitating body image disturbances and emotional distress, which were ameliorated following targeted liposuction. This supports the integration of body-contouring procedures into multidisciplinary care protocols for endocrine-related lipodystrophies, especially when residual physical stigma persists after hormonal normalization [7-9]. 

Body image disorders, such as those secondary to CS or lipodystrophy, significantly impact self-perception, self-esteem, and social functioning. For example, a study by Alcalar et al. (2013) reported that patients with active Cushing’s disease had significantly lower SF-36 scores - particularly in emotional role functioning and mental health domains - compared to controls [10]. Similarly, Akinci et al. (2024) described that patients with partial lipodystrophy demonstrated marked reductions in EQ-5D index values and visual analog scale (VAS) scores, indicating impaired health-related quality of life [9]. These findings underscore that fat redistribution disorders can substantially compromise psychosocial well-being, even after endocrine remission.

This is especially relevant in women, where sociocultural stereotypes surrounding female physical appearance reinforce thinness, symmetry, and youthfulness as standards of personal value and social acceptance [1]. This societal context amplifies body dissatisfaction when visible physical changes occur, even after the clinical remission of endocrine diseases, often leading to social withdrawal, anxiety, or depression [3,10]. Within this framework, plastic surgery - such as reconstructive liposuction - has proven to be a valuable therapeutic tool, offering physical restoration that can enhance self-confidence and promote social reintegration [4]. Postoperative follow-up in our case revealed patient-reported improvements in body image and psychological well-being. While these outcomes were not assessed using formal psychometric tools, the clinical benefit was evident from the patient’s perspective. This aligns with prior findings demonstrating the psychosocial value of reconstructive surgery, which can enhance self-esteem and social reintegration after physical disfigurement [11,12]. These observations underscore the role of plastic surgery not only as a reconstructive intervention, but also as a therapeutic strategy for restoring dignity and quality of life in patients recovering from CS.

Although validated psychometric instruments such as the Body Image Quality of Life Inventory (BIQLI) and the Dysmorphic Concern Questionnaire (DCQ) are available to assess body image disturbances, these were not applied in our case. Nonetheless, they represent useful tools for evaluating subjective impact in both clinical practice and research settings. The BIQLI evaluates the effect of body image on various aspects of life - social interactions, self-worth, sexuality, and emotional well-being - using a Likert scale ranging from -3 (very negative impact) to +3 (very positive impact), providing a quantifiable assessment of its influence on quality of life [5]. The DCQ, on the other hand, identifies dysfunctional concerns about perceived physical flaws by assessing behaviors such as avoidance, mirror checking, and concealment; higher scores are associated with suspected body dysmorphic disorder (BDD) [6]. These tools are useful for initial diagnosis, surgical candidate selection, and postoperative follow-up, as they objectively measure subjective changes related to body image. Their advantages include ease of use, clinical validity, and applicability in research settings. However, they also have limitations: they do not replace comprehensive psychological evaluation, may be influenced by cultural context, and do not detect deeper psychiatric comorbidities. Therefore, a multidisciplinary and ethically grounded approach - integrating plastic surgery, endocrinology, and psychology - is essential to ensure safe and patient-centered treatment planning.

Aesthetic liposuction is associated with significant improvements in perceived body image and patient quality of life [11]. For example, Papadopulos et al. (2019) observed statistically significant increases in perception of one’s own body appearance and high satisfaction with postoperative results [12]. These aesthetic gains were accompanied by psychological improvements: the same study documented an increase in emotional stability and a reduction in postoperative anxiety [12]. Similarly, Kamundi (2023) found that nearly all assessed dimensions of quality of life improved after liposuction (p < 0.05 in most of them). Altogether, these findings suggest that liposuction not only corrects physical alterations typical of CS, but also strengthens self-esteem and psychological well-being by substantially improving satisfaction with one’s body image [11].

Moreover, self-esteem influences adherence to medical treatments and lifestyle changes. By improving self-image through reconstructive surgery, it is plausible that the patient feels more motivated to maintain healthy habits, such as diet and regular exercise, that prevent metabolic relapse [12,13].

Nonetheless, it is important to emphasize that liposuction, in this context, should be viewed as a reconstructive complement, not a primary treatment. There are no established protocols or formal guidelines that explicitly include plastic surgery in the care of cured CS; the decision is personalized, based on the residual functional and psychological impact.

## Conclusions

Reconstructive plastic surgery, though not a primary therapeutic approach for CS, plays a key role in enhancing patients’ quality of life following remission. Liposuction, in particular, offers a safe and effective solution for persistent lipodystrophy, providing aesthetic benefits with minimal scarring, rapid recovery, and low complication rates in properly selected patients.

This case underscores the importance of addressing both physical and psychosocial sequelae after endocrine stabilization. A multidisciplinary approach - encompassing endocrinology, neurosurgery, and plastic surgery - not only restores physical appearance but also contributes to emotional recovery, self-esteem, and overall patient satisfaction.
